# Supplementary material for: Balloon pressure monitoring for radial artery hemostasis after transradial coronary procedures: protocol for a randomized controlled trial
Source: PLoS One. 2026 Jun 4;21(6):e0350563. doi: 10.1371/journal.pone.0350563 (PMC13235883; doi:10.1371/journal.pone.0350563)
Supplement: S2 File — (PDF) [file pone.0350563.s002.pdf]

研究方案

版本号：V1.1　　　　　　日期：2025 年 10 月 11 日

项目名称	气囊压力监测法在桡动脉通畅性止血中的效果：一项随机对照试验		
主要研究者	邹兰	联系方式	13913360716

1. 立题依据

经桡动脉入路（transradial access，TRA）是冠脉介入的首选入路，但桡动脉闭塞（radial artery occlusion，RAO）是最常见的并发症之一。RAO 的危险因子包括不可变因子（如年龄、女性、糖尿病等）和可变因子（如术中危险因子和术后危险因子）。术中危险因子主要包括：反复不成功的桡动脉穿刺、鞘动脉比例增加、术前未用阿司匹林和围术期血管内未用抗凝或剂量过小。而术后危险因子包括特别是阻断性压迫止血、较长时间的压迫止血以及桡动脉痉挛。桡动脉痉挛会增加桡动脉和鞘管及导管间的摩擦力，从而加重内皮损伤。在压迫止血的过程中，始终保持桡动脉的前向血流可有效预防 RAO。

在桡动脉止血中，保持桡动脉血流通畅是预防 RAO 的重要非药物方法，但由于操作的复杂性和需求，全球范围内的采纳率受限，仍有 20%—50% 的患者未能确保血流通畅。因此，探讨替代性技术来增加通畅性止血的成功率尤为重要。

气囊压力监测法通过连接气压显示表，在气囊式桡动脉压迫器的基础上实时显示气囊压力。预实验证明，该方法能精确控制施加的压力，有效避免因压力过高引发的 RAO 和因压力过低导致的出血等并发症。与传统方法相比，它显著提升了患者的安全性和舒适性。

2. 研究内容

2.1 研究目的与意义

探究气囊压力监测法在桡动脉通畅性止血中的效果

2.2 研究内容与设计

本研究为单中心、前瞻性、随机对照优效性试验，旨在评估气囊压力监测法在桡动脉通畅性止血中的效果。试验的优效性界值设定为 3.3%，即气囊压力监测法组的 24 小时桡动脉闭塞（RAO）发生率比标准通畅性止血法组至少低 3.3 个百分点，才能被认为具有临床优效性。

2.3 研究对象

心内科接受冠状动脉造影的患者

2.4 研究步骤

2.4.1 准备阶段

组建由心血管专家、临床研究人员和护士组成的研究团队，并向伦理委员会提交研究方案进行审批，确保研究符合伦理标准。在南京市溧水区人民医院心内科招募并筛选符合条件的患者，并取得他们的知情同意。

2.4.2 随机分组与干预阶段

由独立的数据管理团队使用 SPSS 25.0 软件生成随机数字并将研究对象分为气囊压力监测法组和标准通畅性止血法组。

2.4.2.1 隐蔽分组过程

（1） 生成随机序列

由独立的数据管理团队使用 SPSS 25.0 软件生成随机数字序列，以确保分组的随机性。

（2）分配隐蔽

将生成的随机序列装入按顺序编号、不透光的密封信封中。信封仅在患者完成入组、即将分配干预措施前开启，以防止负责入组和分配的研究人员预先知晓分组情况，从而避免选择偏倚。

（3） 样本量计算

通过查阅高质量文献得出对照组 24 小时 RAO 发生率为 4.3%，考虑到优效性界值为 3.3%（即干预组的事件率应比对照组低 3.3 个百分点），采用 PASS15.0.5 软件计算样本量，设双侧 α=0.05，把握度为 80%，结果得到两组样本总量为 736 例。考虑退出情况，按照至少 10% 计算，我们计划入组 818 例患者。

（4）分组实施

在手术开始前，由独立的研究协调员根据封闭的随机序列进行分组，将研究对象分为气囊压力监测法组和标准通畅性止血法组。

（5）记录与保密

所有分组信息将被保存在安全的档案中，并仅限于研究协调员和统计人员查看，以确保数据的保密性和研究的公正性。

2.4.2.2 干预阶段

2.4.2.2.1 气囊压力监测法

在气囊式桡动脉压迫器的基础上，连接气压显示表，从而精准显示气囊压力。

（1） 首次充气方法

手术结束时，使用气囊式压迫装置，并通过三通连接气压显示表，将气囊充气至 260mmHg。拔除鞘管后，立即降低气囊压力至有穿刺点渗血，随后增加 20mmHg 气压，观察穿刺点是否继续出血。出血停止后记录当前气囊压力值，若仍有出血，继续增加 20mmHg 压力，直至停止，并记录压力值。

（2） 术后 30 分钟放气方法

将气囊压力调整至 60mmHg。如有出血，增加 20mmHg，观察穿刺点出血情况。停止出血后记录当前气囊压力值，如仍有出血，继续增加 20mmHg 压力，直至停止出血，并记录压力值。

（3）术后 60 分钟、90 分钟和 120 分钟分步骤放气方法

在这三个时间点每次放气，先降低气囊压力 30mmHg。如果穿刺点出现出血，立即增加气囊压力 20mmHg，并观察出血情况。一旦出血停止，记录当前气囊压力值。如果仍然出血，重复增加 20mmHg，直到出血完全停止，每次更新并记录压力值。如果气囊压力降到零，需保留止血带至手术结束后 24 小时。在这个过程中，提醒患者注意避免任何可能对穿刺部位施加过度压力的活动，比如支撑或过度使用穿刺的上肢。

2.4.2.2.2 标准通畅性止血法

（1） 首次充气方法

使用气囊式桡动脉压迫器放置在引入器鞘的入口部位，用 18ml 空气充气，直至达到初步止血。然后取出导管鞘，降低气压，直到穿刺部位出现搏动性血液。之后，加入 1—2ml 空气，证实无出血，观察食指血氧饱和度及脉搏曲线。随后立即手动压迫尺动脉 2 分钟，观察血氧饱和度和脉搏曲线 （反向 Barbeau 试验）。当血氧饱和度 > 90% 且脉冲曲线足够时，则操作结束。未实现通畅止血的情况下，每 15 分钟重复一次该方案，直到读数上升至 90% 或以上。

（2）术后 30 分钟放气方法

抽出气囊内气体 1ml。

（3） 术后 60 分钟放气方法

抽出气囊内气体 3ml。

（4） 术后 90 分钟放气方法

抽出气囊内气体 3ml。

（5）术后 120 分钟放气方法

移除止血带。

在放气过程中，若有出血，再次充入适量气体直至不出血，并记录。

2.4.3 偏倚控制与盲法实施

鉴于两种干预措施（气囊压力监测法 vs. 标准通畅性止血法）的特性截然不同，对执行止血操作的人员（操作者）及患者实施盲法不可行。因此，本研究为一项开放标签试验。为最大程度减少评估偏倚和测量偏倚，本研究实施以下盲法措施：

（1） 终点评估者盲法

主要终点（术后 24 小时桡动脉闭塞）由一名不知晓患者分组情况的独立研究者使用多普勒超声进行评估。

（2） 数据分析盲法

最终的统计分析将由一名不知晓患者分组情况的独立统计学家完成。

（3） 揭盲程序

由于主治医师本身不设盲，因此无需为临床管理进行紧急揭盲。终点评估者和统计分析人员的盲态将在整个试验期间保持。若因数据安全监查委员会（DSMB）进行安全性评估或监管要求等特殊情况，必须对某位参与者的分组进行揭盲，需向试验指导委员会提交正式申请并获得批准。揭盲将由持有分配清单的独立统计学家执行，并完整记录揭盲日期及原因。

2.4.4 术后观察与数据采集阶段

在术后 30 分钟、60 分钟、90 分钟和 120 分钟对患者进行观察，记录出血情况和气囊压力值，并收集相关数据。

2.4.5 数据处理与统计分析方法

（1） 基线特征比较

在开始主要分析之前，应进行组间基线特征的比较，包括但不限于年龄、性别、BMI、病史等，以验证随机分组的均衡性。根据变量的类型，采用适当的统计方法，如对连续变量使用独立样本 t 检验或 Mann-Whitney U 检验，对分类变量使用卡方检验或 Fisher 精确检验，确保两组在基线特征上的可比性。

（2） 主要终点分析

对于 24 小时 RAO 发生率，采用卡方检验或 Fisher 精确检验比较两组间的差异。优效性界值设定为 3.3%，即气囊压力监测法组的 RAO 发生率比标准通畅性止血法组至少低 3.3 个百分点，才能被认为具有临床优效性。此外，计算比值比（OR）及其 95% 置信区间（CI），以量化两组间的相对风险差异。

（3） 次要终点分析

对于血管并发症和出血并发症的发生率，同样采用卡方检验或 Fisher 精确检验，并计算相应的 OR 和 CI，评估两组在次要终点上的差异。

（4） 便捷性分析

对于便捷性指标，如总时间和操作人员数量，使用独立样本 t 检验或 Mann-Whitney U 检验，比较两组在操作效率上的差异。

（5）多元逻辑回归分析 1

在气囊压力监测组中，将 RAO 发生作为因变量，纳入气囊压力、压迫时间及患者的基线特征（如年龄、性别、糖尿病、出血史、管鞘直径等）作为自变量，建立多元逻辑回归模型。首先进行单变量分析，筛选出具有统计学意义的变量，然后将这些变量纳入多元模型中。使用 Hosmer-Lemeshow 检验评估模型拟合度，确保模型对数据的拟合适当。基于 OR 值，解释首次压迫压力和压迫时间对 RAO 发生的影响。

（6） 多元逻辑回归分析 2

在气囊压力监测组中，将出血并发症发生作为因变量，纳入气囊压力、压迫时间及患者的基线特征（如年龄、性别、糖尿病、RAO 史、管鞘直径等）作为自变量，建立多元逻辑回归模型。同样，首先进行单变量分析，筛选出具有统计学意义的变量，然后将这些变量纳入多元模型中。使用 Hosmer-Lemeshow 检验评估模型拟合度。基于 OR 值，解释首次压迫压力和压迫时间对出血发生的影响。

（7）亚组分析

对于气囊压力监测组，根据气囊压力和压迫时间进行分层，比较不同压力和不同压迫时间亚组间的差异。使用卡方检验、Fisher 精确检验或多元逻辑回归分析，探讨各亚组在 RAO 发生率和出血发生率上的差异。

2.4.6 研究总结与后续工作

总结研究结果，评估气囊压力监测法的效果及其与标准止血方案的对比，探讨其在临床实践中的应用前景，并关注患者的长期随访结果，以优化止血方法和评估对远期预后的影响。

2.4.7 操作者培训与质量控制

由于操作者未设盲，为减少操作偏倚和检测偏倚，将实施以下严格的培训和质控措施：

操作者资格：所有直接执行气囊压力监测或评估止血效果的人员均须具备相应的临床资质和经验。

标准化培训：试验开始前，所有相关人员须完成集中培训，内容涵盖研究方案、设备操作流程、终点事件识别与管理、病例报告表填写规范等。

资格认证：培训后，操作者需在主要研究者或指定督导人员监督下，成功完成至少 3 例模拟或真实患者的全流程操作，经确认能准确、一致地执行方案后方可获得正式操作资格。

持续质量监控：主要研究者将定期审核操作记录与数据，确保方案依从性。发生任何与操作相关的不良事件将进行复盘，必要时重新培训。

2.5 评价指标

2.5.1 主要终点

24 小时 RAO 发生率。通过超声检查确认桡动脉通畅状态，并计算 RAO 发生率。

2.5.2 次要终点

2.5.2.1 血管并发症发生率

包括桡动脉穿孔、动静脉瘘和假性动脉瘤，计算方法为发生相关并发症人数占总患者数量的百分比。

2.5.2.2 出血并发症发生率

包括重大出血、轻微出血和血肿，计算方法为发生相关并发症人数占总患者数量的百分比。

2.5.2.3 便捷性

（1）指标

a. 总时间：从拔除动脉鞘至完全移除止血装置并确认穿刺点止血的累积时间，仅统计主动操作和评估时间（排除 30/60/90 分钟固定观察期）。“首次充气” 指的是从设备准备好到充气完成的时间，“术后各时间点的放气及调整” 包括每次放气及调整的时间。

b. 操作人员数量：每种方法所需的操作人员数量。

（2）计算方法

a.总时间：分别记录每种方法完成整个止血过程的总时间（分钟）。

b.操作人员数量：记录在整个过程中实际需要的操作人员数量。

2.6 数据管理与统计分析计划、资料保密计划

2.6.1 数据管理与统计分析计划

数据管理将通过标准化的数据收集表格和电子数据采集系统来完成，确保试验数据的准确性和完整性。数据监控团队将实时检查数据的质量，处理任何异常情况，并进行验证以确保数据的真实性。统计分析将应用描述性和推断性统计方法，进行效力分析以确定样本量的合理性，并根据预设流程进行数据清洗和分析，最终通过图表和文字报告展示结果，确保分析的透明性和可靠性。

2.6.2 资料保密计划

试验将采取严格的保密措施，所有参与者均需签署保密协议并接受保密培训，确保其了解并遵守保密规定。试验数据和材料将分类存储，设定访问权限，仅限授权人员访问。同时，试验场所和数据存储系统将进行严格管理，防止数据泄露。应急响应计划将应对可能的保密泄露事件，确保迅速采取补救措施并通知相关人员，以维护试验的保密性和完整性。

2.7 技术路线

技术路线图见下图：

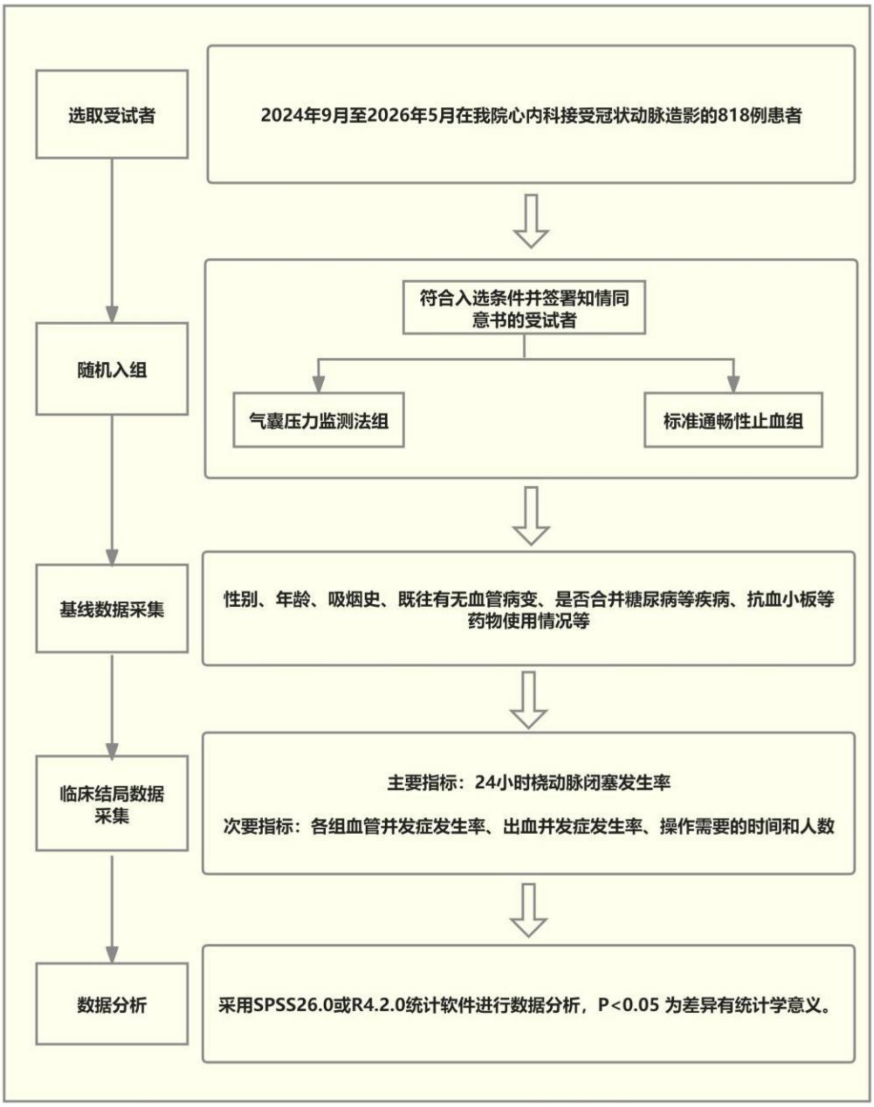

3. 受试者招募

3.1 纳入标准

（1） 年龄≥18 岁，且 < 80 岁；

（2） 在心内科接受冠状动脉造影的患者；

（3） 意识清晰，有一定的理解和表达能力的患者；

（4） 愿意参加研究并提供书面知情同意书的患者；

（5） 抗血小板药的使用：术前一天阿司匹林肠溶片 300mg + 替格瑞洛片 180mg / 硫酸氢氯吡格雷片 300mg；手术当天阿司匹林肠溶片 100mg + 替格瑞洛片 90mg / 硫酸氢氯吡格雷片 75mg。

3.2 排除标准

（1） 上肢残疾或畸形患者；

（2） 局部皮肤或组织水肿或感染患者；

（3） 凝血功能异常患者或严重合并症患者、2 次或 2 次以上同侧桡动脉穿刺患者；

（4） 穿刺点非掌横纹下方 1—2cm。

3.3 退出和终止标准

3.3.1 患者退出标准

（1） 不愿继续参与：患者在试验过程中主动选择退出，因任何个人原因（如不适、时间冲突等）；不符合继续参与的条件：患者在试验期间出现新的健康问题或并发症，导致不再符合研究的纳入标准。

（2） 严重不良事件：患者出现与试验相关的严重不良事件（如严重的穿刺部位感染等），影响其继续参与的安全性或研究结果的可靠性。

（3） 未遵守试验方案：患者未能按照研究要求完成指定的程序或治疗，如未能按时进行术后观察或数据记录。

3.3.2 试验终止标准

（1） 试验安全性问题：如果在试验过程中发现安全性问题，例如气囊压力监测法组的严重不良事件发生率显著高于标准通畅性止血组，需要终止试验以保护患者安全。

（2） 数据质量问题：若发现数据收集或管理过程中存在重大缺陷，影响数据的可靠性和有效性，可能导致试验终止。

（3） 伦理问题：如发现试验过程中的伦理问题或对患者权益的保护不足，需终止试验并进行相应的整改。

（4） 试验目标未达成：在试验进行过程中，若经分析发现试验无法有效地回答研究假设或无法实现研究目标，终止试验。

3.4 研究参与者参加研究的时间（每次参与时间与总时间）

- 基线评估：约 1 小时，包括临床评估和实验室检查等，确认研究参与资格。
- 第一次干预：4-6 小时（包括手术和监测）；实施气囊压力监测法或标准止血方法。
- 术后评估：约 10 分钟；评估恢复情况及 24 小时桡动脉闭塞情况。

3.5 招募过程

3.5.1 招募程序及启动时间

招募程序将从伦理审批通过后正式启动，预计开始时间为 2024 年 9 月 1 日。招募将分为几个阶段，包括初期筛选、资格确认，以及最终登记。心内科医生将根据纳入标准和排除标准对患者进行筛选，确认符合条件后，将向患者介绍试验并获取知情同意。

3.5.2 招募方式

- 医院内招募：在南京市溧水区人民医院心内科内张贴招募通知，向所有接受冠状动脉血管造影的冠心病患者介绍试验信息。
- 医生推荐：由心内科医生主动推荐符合条件的患者，并向其详细说明试验的目的和流程。
- 患者信息管理系统：利用医院的患者信息管理系统筛选符合条件的患者，并向他们发送招募邀请函。

3.5.3 预期招募人数

预期招募 818 名患者，按照随机分组的设计，将其分为气囊压力监测法组和标准通畅性止血组，各组各 409 人。招募人数将根据试验进展和实际情况进行调整，以确保研究的统计效力。

3.5.4 是否有补偿

参与本研究的患者不会因参与研究而获得任何经济补偿。

4. 风险 / 获益评估

4.1 获益（个人和社会获益）

4.1.1 个人获益

患者参与试验将有机会接受最新的医疗技术和治疗方案，如气囊压力监测法可能提高止血效果，降低术后并发症的风险。试验过程中的详细检查和监测将帮助及早发现潜在健康问题，提升患者的整体健康管理。

4.1.2 社会获益

试验结果将为心内科接受冠状动脉造影患者的治疗和止血方法的优化提供重要数据，可能对心内科领域的临床实践产生积极影响。通过比较气囊压力监测法与传统止血方法的效果，试验有助于改进医疗技术，提升患者的治疗效果和生活质量。最终，这些研究成果将有助于推动心血管疾病的治疗进步，降低相关的公共健康负担。

4.2 风险

4.2.1 可能的风险

（1） 操作风险：使用气囊压力监测法可能引发的并发症，包括气囊压力失控、血管损伤等。

（2） 术后并发症：患者可能会出现术后并发症，如出血、血肿、桡动脉穿孔、动静脉瘘等。尽管研究旨在优化止血方法，但这些风险仍需密切监测和及时处理。

（3） 个体差异：不同患者对气囊压力监测法的耐受性可能有所不同，特别是老年人、孕妇及有严重基础疾病的患者，可能面临更高的并发症风险。

（4） 数据隐私风险：患者的个人信息和健康数据需要严格保护，任何数据泄露都可能造成隐私侵犯。

（5） 伦理风险：确保知情同意的充分性和患者对研究的理解是必要的，尤其是在特殊人群中。任何不充分的知情同意可能会引发伦理问题。

（6） 试验风险：试验过程中可能会发现新的未预见的风险，需要及时调整研究方案，以保障患者的安全。

4.2.2 风险最小化措施

（1） 严格的医疗操作规程：确保气囊压力监测法的使用符合标准化操作规程，并由经过专门培训的医务人员进行。

（2） 定期监测与评估：试验期间对患者进行定期健康监测和评估，及时发现并处理可能出现的风险。

（3） 充分的知情同意：在试验开始前，向患者详细解释所有可能的风险，并确保他们理解和同意参与试验。

（4） 应急处理机制：制定详细的应急处理计划，确保在出现不良事件时能够迅速采取行动，以减少对患者的影响。

4.3 特殊人群保护的风险及保护

4.3.1 特殊人群保护的风险

（1） 老年人：老年患者可能存在更高的并发症风险，如心血管疾病或多重慢性病，且对新的医疗技术的耐受性可能较差。

（2） 孕妇及哺乳期女性：此类人群可能对试验中的医疗措施有特殊需求，可能对药物或治疗产生不良反应。

（3） 患有严重基础疾病的患者：这些患者可能对试验干预的耐受性较差，风险较高。

4.3.2 特殊人群保护的保护措施

（1） 严格筛选和评估：对特殊人群进行更为严格的筛选和健康评估，确保他们符合参与试验的条件，并能承受试验过程中的风险。

（2） 个性化风险管理：为特殊人群制定个性化的风险管理计划，包括定期的健康检查和调整治疗方案。

（3） 伦理审查和咨询：在试验设计阶段充分考虑特殊人群的需求，获得伦理委员会的审查和批准，并提供适当的咨询和支持。

（4） 充分沟通与同意：确保特殊人群的患者在参与试验前充分了解所有相关风险，并提供详细的知情同意书，确保他们自愿参与并能做出明智的决定。

5. 研究中的伦理问题

5.1 知情同意

知情同意是医疗研究中的基本伦理原则。在招募参与者时，确保他们充分理解试验的目的、过程、潜在风险和获益至关重要。研究团队将提供详细的说明书，确保患者有足够的时间进行咨询，回答他们的疑问，并自愿作出参与决定。特别是对特殊人群（如老年人、孕妇和有严重基础疾病的患者），需额外关注其理解能力和决策权。

5.2 隐私保护

在试验期间，涉及患者的个人信息及健康数据必须得到严格保护，确保符合数据隐私和保护的相关法律法规。所有参与者的数据应匿名处理，非授权人员不得接触敏感信息。研究团队将采取必要的技术和管理措施，确保数据的安全性和保密性。

5.3 风险与获益的平衡

在研究进行中，必须确保参与者面临的风险与其可能获得的获益相对平衡。研究设计应明确评估风险程度，并进行严格的监测。如果在试验过程中观察到不良事件或风险增加，研究团队需及时调整研究方案，保护参与者的安全。

5.4 资源分配公平性

在招募过程中，应确保所有符合条件的患者都有公平的参与机会。避免因患者的社会经济状态、种族、性别等因素而导致参与权的歧视。研究团队将采取主动措施，确保招募过程透明，并公平对待所有潜在参与者。

5.5 不良事件的处理

发生任何与试验相关的不良事件时，需立即进行记录和报告，并采取适当的干预措施。研究团队应确保参与与者得到及时的医疗救助，并在试验过程中保持对其健康状况的持续关注。

5.6 伦理审查与监控

实施伦理审查委员会（IEC）或机构审查委员会（IRB）的审核，并在整个研究过程中进行定期监控，确保研究始终遵循伦理标准和法规要求。

6. 年度计划

- 2024 年 9 月 - 2026 年 5 月：完成 818 例患者的筛选及入组，年中召开项目组工作汇报会，总结研究进行中遇到的问题，并适当调整研究内容与进度，进一步完善研究方案。
- 2026 年 6 月 - 2026 年 12 月：汇总各项数据，进行统计学分析，撰写论文和结题报告。

申请人承诺并签字：

我保证申请内容的真实性。我将履行课题负责人的职责，严格遵守国家有关临床研究的相关规定，切实保证研究工作的时间，认真开展工作，按时报送有关材料，自觉按照伦理委员会批件的要求递交研究过程中的相关报告。若填报失实和违反规定，本人将承担全部责任。

课题负责人签字：

日　期：

Research Protocol

Version No.: V1.1 Date: October 11, 2025

Project Name	Efficacy of Balloon Pressure Monitoring Method in Patent Hemostasis of the Radial Artery: A Randomized Controlled Trial		
Principal Investigator	Lan Zou	Contact Information	13913360716

1. Rationale for the Study

Transradial access (TRA) is the preferred approach for coronary intervention, but radial artery occlusion (RAO) is one of the most common complications. Risk factors for RAO include non-modifiable factors (such as age, female gender, diabetes mellitus, etc.) and modifiable factors (such as intraoperative and postoperative risk factors). Intraoperative risk factors mainly include: repeated unsuccessful radial artery punctures, increased sheath-to-artery ratio, preoperative non-use of aspirin, and intraoperative inadequate anticoagulation or suboptimal dosage. Postoperative risk factors include, in particular, occlusive compression hemostasis, prolonged compression hemostasis, and radial artery spasm. Radial artery spasm increases the friction between the radial artery, sheath, and catheter, thereby exacerbating endothelial injury. Maintaining antegrade blood flow in the radial artery during compression hemostasis can effectively prevent RAO.

In radial artery hemostasis, maintaining radial artery patency is an important non-pharmacological method to prevent RAO. However, due to the complexity and requirements of the operation, the global adoption rate is limited, and 20%—50% of patients still fail to ensure patency. Therefore, exploring alternative technologies to increase the success rate of patent hemostasis is particularly important.

The balloon pressure monitoring method displays real-time balloon pressure on the basis of a balloon-type radial artery compression device by connecting a pressure gauge. Preliminary experiments have shown that this method can precisely control the applied pressure, effectively avoiding complications such as RAO caused by excessive pressure and bleeding caused by insufficient pressure. Compared with traditional methods, it significantly improves patient safety and comfort.

2. Study Content

2.1 Research Objectives and Significance

To explore the efficacy of the balloon pressure monitoring method in patent hemostasis of the radial artery.

2.2 Research Content and Design

This is a single-center, prospective, randomized controlled superiority trial aimed at evaluating the efficacy of the balloon pressure monitoring method in patent hemostasis of the radial artery. The superiority margin of the trial is set at 3.3%, meaning that the 24-hour RAO incidence in the balloon pressure monitoring group must be at least 3.3 percentage points lower than that in the standard patent hemostasis group to be considered clinically superior.

2.3 Study Participants

Patients undergoing coronary angiography in the Department of Cardiology.

2.4 Study Procedures

2.4.1 Preparation Phase

Establish a research team consisting of cardiovascular experts, clinical researchers, and nurses, and submit the research protocol to the Ethics Committee for approval to ensure the study complies with ethical standards. Recruit and screen eligible patients in the Department of Cardiology, Nanjing Lishui District People's Hospital, and obtain their informed consent.

2.4.2 Randomization and Intervention Phase

An independent data management team will generate random numbers using SPSS 25.0 software and divide the study participants into the balloon pressure monitoring group and the standard patent hemostasis group.

2.4.2.1 Concealed Allocation Process

(1) Generation of Random Sequence

An independent data management team will generate a random number sequence using SPSS 25.0 software to ensure the randomness of grouping.

(2) Allocation Concealment

The generated random sequence will be placed in sequentially numbered, opaque sealed envelopes. The envelopes will only be opened after the patient has completed enrollment and is about to receive the intervention to prevent researchers responsible for enrollment and allocation from knowing the grouping in advance, thereby avoiding selection bias.

(3) Sample Size Calculation

By reviewing high-quality literature, the 24-hour RAO incidence in the control group is 4.3%. Considering the superiority margin of 3.3% (i.e., the event rate in the intervention group should be 3.3 percentage points lower than that in the control group), PASS 15.0.5 software is used for sample size calculation. With a two-sided $\alpha=0.05$ and power of 80%, the total

sample size for the two groups is 736 cases. Considering attrition, calculated at least 10%, we plan to enroll 818 patients.

(4) Implementation of Grouping

Before the start of surgery, an independent study coordinator will perform grouping according to the sealed random sequence, dividing the study participants into the balloon pressure monitoring group and the standard patent hemostasis group.

(5) Documentation and Confidentiality

All grouping information will be stored in secure files and accessible only to the study coordinator and statisticians to ensure data confidentiality and research impartiality.

2.4.2.2 Intervention Phase

2.4.2.2.1 Balloon Pressure Monitoring Method

On the basis of a balloon-type radial artery compression device, a pressure gauge is connected to accurately display the balloon pressure.

(1) Initial Inflation Method

At the end of the operation, use a balloon compression device and connect a pressure gauge through a three-way stopcock to inflate the balloon to 260 mmHg. Immediately after removing the sheath, reduce the balloon pressure until oozing occurs at the puncture site, then increase the pressure by 20 mmHg and observe if bleeding continues. Record the current balloon pressure when bleeding stops; if bleeding persists, continue increasing the pressure by 20 mmHg until bleeding ceases, and record the pressure value.

(2) Deflation Method at 30 Minutes Postoperatively

Adjust the balloon pressure to 60 mmHg. If bleeding occurs, increase the pressure by 20 mmHg and observe the bleeding at the puncture site. Record the current balloon pressure when bleeding stops; if bleeding persists, continue increasing the pressure by 20 mmHg until bleeding ceases, and record the pressure value.

(3) Stepwise Deflation Methods at 60 Minutes, 90 Minutes, and 120 Minutes Postoperatively

At each of these three time points, first reduce the balloon pressure by 30 mmHg. If bleeding occurs at the puncture site, immediately increase the balloon pressure by 20 mmHg and observe the bleeding. Once bleeding stops, record the current balloon pressure. If bleeding still persists, repeat the 20 mmHg increase until bleeding is completely controlled, updating and recording the pressure value each time. If the balloon pressure drops to zero, retain the tourniquet until 24 hours after the surgery. During this process, remind the patient to avoid any activities that may exert excessive pressure on the puncture site, such as supporting or overusing the punctured upper limb.

2.4.2.2.2 Standard Patent Hemostasis Method

(1) Initial Inflation Method

Place a balloon-type radial artery compression device at the entry site of the introducer sheath, inflate with 18 ml of air until initial hemostasis is achieved. Then remove the catheter sheath, reduce the air pressure until pulsatile blood appears at the puncture site. Subsequently, add 1—2 ml of air to confirm no bleeding, and observe the index finger oxygen saturation and pulse curve. Immediately after, manually compress the ulnar artery for 2 minutes and observe the oxygen saturation and pulse curve (reverse Barbeau test). The operation is completed when the oxygen saturation > 90% and the pulse curve is adequate. If patent hemostasis is not achieved, repeat the protocol every 15 minutes until the reading rises to 90% or above.

(2) Deflation Method at 30 Minutes Postoperatively

Withdraw 1 ml of gas from the balloon.

(3) Deflation Method at 60 Minutes Postoperatively

Withdraw 3 ml of gas from the balloon.

(4) Deflation Method at 90 Minutes Postoperatively

Withdraw 3 ml of gas from the balloon.

(5) Deflation Method at 120 Minutes Postoperatively

Remove the tourniquet.

During the deflation process, if bleeding occurs, re-inflate with an appropriate amount of gas until bleeding stops, and record the details.

2.4.3 Bias Control and Blinding Implementation

Given the distinct characteristics of the two interventions (balloon pressure monitoring method vs. standard patent hemostasis method), blinding of the operators performing the hemostasis and the patients is not feasible. Therefore, this study is an open-label trial. To minimize assessment bias and measurement bias to the greatest extent, the following blinding measures are implemented in this study:

(1) Outcome Assessor Blinding

The primary endpoint (24-hour RAO) will be assessed by an independent researcher unaware of the patient's grouping using Doppler ultrasound.

(2) Data Analyst Blinding

The final statistical analysis will be performed by an independent statistician unaware of the patient's grouping.

(3) Unblinding Procedure

Since the attending physicians are not blinded, emergency unblinding for clinical management is not required. The blinding of outcome assessors and statisticians will be maintained throughout the trial. If unblinding of a participant's grouping is necessary for special circumstances such as safety assessment by the Data Safety Monitoring Board (DSMB) or regulatory requirements, a formal application must be submitted to the Trial Steering Committee and approved. Unblinding will be performed by an independent statistician holding the allocation list, and the date and reason for unblinding will be fully documented.

2.4.4 Postoperative Observation and Data Collection Phase

Observe the patients at 30 minutes, 60 minutes, 90 minutes, and 120 minutes postoperatively, record bleeding and balloon pressure values, and collect relevant data.

2.4.5 Data Processing and Statistical Analysis Methods

(1) Comparison of Baseline Characteristics

Before conducting the primary analysis, comparisons of baseline characteristics between groups should be performed, including but not limited to age, gender, BMI, medical history, etc., to verify the balance of randomization. Appropriate statistical methods will be used according to the type of variables: independent samples t-test or Mann-Whitney U test for continuous variables, and Chi-square test or Fisher's exact test for categorical variables, to ensure the comparability of the two groups in terms of baseline characteristics.

(2) Primary Endpoint Analysis

For the 24-hour RAO incidence, Chi-square test or Fisher's exact test will be used to compare differences between the two groups. The superiority margin is set at 3.3%, meaning that the RAO incidence in the balloon pressure monitoring group must be at least 3.3 percentage points lower than that in the standard patent hemostasis group to be considered clinically superior. In addition, the odds ratio (OR) and its 95% confidence interval (CI) will be calculated to quantify the relative risk difference between the two groups.

(3) Secondary Endpoint Analysis

For the incidence of vascular complications and bleeding complications, Chi-square test or Fisher's exact test will also be used, and the corresponding OR and CI will be calculated to evaluate differences between the two groups in secondary endpoints.

(4) Convenience Analysis

For convenience indicators such as total time and number of operators, independent samples t-test or Mann-Whitney U test will be used to compare differences in operational efficiency between the two groups.

(5) Multivariate Logistic Regression Analysis 1

In the balloon pressure monitoring group, RAO occurrence will be taken as the dependent variable, and balloon pressure, compression time, and patient baseline characteristics (such as age, gender, diabetes mellitus, bleeding history, sheath diameter, etc.) will be included as independent variables to establish a multivariate logistic regression model. Univariate analysis will first be performed to screen for statistically significant variables, which will then be included in the multivariate model. The Hosmer-Lemeshow test will be used to assess model fit to ensure the model appropriately fits the data. Based on the OR values, the effects of initial compression pressure and compression time on RAO occurrence will be explained.

(6) Multivariate Logistic Regression Analysis 2

In the balloon pressure monitoring group, bleeding complication occurrence will be taken as the dependent variable, and balloon pressure, compression time, and patient baseline characteristics (such as age, gender, diabetes mellitus, RAO history, sheath diameter, etc.) will be included as independent variables to establish a multivariate logistic regression model. Similarly, univariate analysis will first be performed to screen for statistically significant variables, which will then be included in the multivariate model. The Hosmer-Lemeshow test will be used to assess model fit. Based on the OR values, the effects of initial compression pressure and compression time on bleeding occurrence will be explained.

(7) Subgroup Analysis

In the balloon pressure monitoring group, stratification will be performed according to balloon pressure and compression time, and differences between subgroups with different pressures and compression times will be compared. Chi-square test, Fisher's exact test, or multivariate logistic regression analysis will be used to explore differences in RAO incidence and bleeding incidence among various subgroups.

2.4.6 Study Summary and Follow-up Work

Summarize the study results, evaluate the efficacy of the balloon pressure monitoring method compared with the standard hemostasis protocol, explore its application prospects in clinical practice, and pay attention to the long-term follow-up results of patients to optimize hemostasis methods and assess the impact on long-term prognosis.

2.4.7 Operator Training and Quality Control

Since operators are not blinded, the following strict training and quality control measures will be implemented to reduce operational bias and detection bias:

Operator Qualifications: All personnel directly performing balloon pressure monitoring or assessing hemostasis efficacy must possess corresponding clinical qualifications and experience.

Standardized Training: Before the start of the trial, all relevant personnel must complete centralized training covering the research protocol, equipment operation procedures, identification and management of endpoint events, and case report form filling standards.

Qualification Certification: After training, operators must successfully complete the full-process operation of at least 3 simulated or real patients under the supervision of the

principal investigator or designated supervisor, and obtain formal operation qualifications only after confirming their ability to execute the protocol accurately and consistently.

Continuous Quality Monitoring: The principal investigator will regularly review operation records and data to ensure protocol compliance. Any adverse events related to operations will be reviewed, and retraining will be conducted if necessary.

2.5 Evaluation Indicators

2.5.1 Primary Endpoint

24-hour RAO incidence. The patency status of the radial artery will be confirmed by ultrasound, and the RAO incidence will be calculated.

2.5.2 Secondary Endpoints

2.5.2.1 Incidence of Vascular Complications

Including radial artery perforation, arteriovenous fistula, and pseudoaneurysm. The calculation method is the percentage of patients with relevant complications among the total number of patients.

2.5.2.2 Incidence of Bleeding Complications

Including major bleeding, minor bleeding, and hematoma. The calculation method is the percentage of patients with relevant complications among the total number of patients.

2.5.2.3 Convenience

(1) Indicators

a. Total Time: Cumulative time from sheath removal to complete removal of the hemostatic device and confirmation of hemostasis at the puncture site, including only active operation and assessment time (excluding fixed observation periods of 30/60/90 minutes). "Initial inflation" refers to the time from equipment preparation to completion of inflation, and "deflation and adjustment at each postoperative time point" includes the time for each deflation and adjustment.

b. Number of Operators: The number of operators required for each method.

(2) Calculation Methods

a. Total Time: Record the total time (in minutes) to complete the entire hemostasis process for each method separately.

b. Number of Operators: Record the actual number of operators required throughout the process.

2.6 Data Management and Statistical Analysis Plan, Data Confidentiality Plan

2.6.1 Data Management and Statistical Analysis Plan

Data management will be completed through standardized data collection forms and an electronic data capture system to ensure the accuracy and completeness of trial data. The data monitoring team will conduct real-time quality checks of the data, handle any abnormalities, and perform verification to ensure data authenticity. Descriptive and inferential statistical methods will be applied for statistical analysis, including power analysis to determine the rationality of the sample size. Data cleaning and analysis will be performed according to preset procedures, and results will be presented through charts and written reports to ensure transparency and reliability of the analysis.

2.6.2 Data Confidentiality Plan

The trial will adopt strict confidentiality measures. All participants must sign a confidentiality agreement and receive confidentiality training to ensure they understand and comply with confidentiality regulations. Trial data and materials will be stored in categories with access permissions set, limited to authorized personnel only. Meanwhile, the trial site and data storage system will be strictly managed to prevent data leakage. An emergency response plan will address potential confidentiality breaches to ensure prompt remedial measures are taken and relevant personnel are notified to maintain the confidentiality and integrity of the trial.

2.7 Technical Route

The technical route is shown in the figure below:

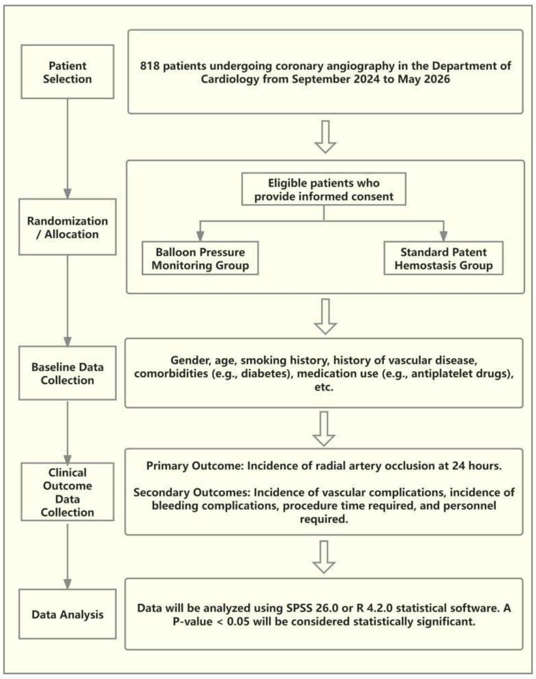

3. Recruitment of Study Participants

3.1 Inclusion Criteria

(1) Aged ≥18 years and <80 years;

(2) Patients undergoing coronary angiography in the Department of Cardiology;

(3) Patients with clear consciousness and certain understanding and expression abilities;

(4) Patients willing to participate in the study and provide written informed consent;

(5) Use of antiplatelet drugs: Aspirin enteric-coated tablets 300 mg + Ticagrelor tablets 180 mg / Clopidogrel bisulfate tablets 300 mg one day before surgery; Aspirin enteric-coated tablets 100 mg + Ticagrelor tablets 90 mg / Clopidogrel bisulfate tablets 75 mg on the day of surgery.

3.2 Exclusion Criteria

(1) Patients with upper limb disability or deformity;

(2) Patients with local skin or tissue edema or infection;

(3) Patients with coagulopathy, severe comorbidities, or those who have undergone 2 or more ipsilateral radial artery punctures;

(4) Puncture site not 1—2 cm below the palmar crease.

3.3 Withdrawal and Termination Criteria

3.3.1 Patient Withdrawal Criteria

(1) Unwillingness to continue participation: Patients voluntarily choose to withdraw during the trial for any personal reasons (such as discomfort, time conflict, etc.); Failure to meet the conditions for continued participation: Patients develop new health problems or complications during the trial, making them no longer eligible for the study.

(2) Severe adverse events: Patients experience trial-related severe adverse events (such as severe puncture site infection), which affect the safety of their continued participation or the reliability of the study results.

(3) Non-compliance with the trial protocol: Patients fail to complete the specified procedures or treatments as required by the study, such as failing to attend postoperative follow-up or data recording on time.

3.3.2 Trial Termination Criteria

(1) Trial safety issues: If safety problems are identified during the trial, such as a significantly higher incidence of severe adverse events in the balloon pressure monitoring group than in the standard patent hemostasis group, the trial needs to be terminated to protect patient safety.

(2) Data quality issues: If major flaws are found in the data collection or management process that affect the reliability and validity of the data, the trial may be terminated.

(3) Ethical issues: If ethical issues are identified during the trial or insufficient protection of patient rights and interests is found, the trial must be terminated and corresponding rectifications made.

(4) Failure to achieve trial objectives: If during the trial, analysis shows that the trial cannot effectively answer the research hypothesis or achieve the research objectives, the trial will be terminated.

3.4 Duration of Study Participation (Time per Participation and Total Time)

• Baseline assessment: Approximately 1 hour, including clinical assessment and laboratory tests to confirm eligibility for study participation.

• First intervention: 4-6 hours (including surgery and monitoring); Implementation of the balloon pressure monitoring method or standard hemostasis method.

• Postoperative assessment: Approximately 10 minutes; Assessment of recovery status and 24-hour radial artery occlusion.

3.5 Recruitment Process

3.5.1 Recruitment Procedures and Start Time

The recruitment process will officially start after ethical approval, with an expected start date of September 1, 2024. Recruitment will be divided into several phases, including initial screening, eligibility confirmation, and final registration. Cardiologists will screen patients according to the inclusion and exclusion criteria, and after confirming eligibility, introduce the trial to the patients and obtain informed consent.

3.5.2 Recruitment Methods

• Hospital-based recruitment: Post recruitment notices in the Department of Cardiology, Nanjing Lishui District People's Hospital, and introduce trial information to all coronary heart disease patients undergoing coronary angiography.

• Physician recommendation: Cardiologists actively recommend eligible patients and explain the purpose and process of the trial in detail.

• Patient information management system: Use the hospital's patient information management system to screen eligible patients and send them recruitment invitations.

3.5.3 Expected Recruitment Number

It is expected to recruit 818 patients, who will be randomly divided into the balloon pressure monitoring group and the standard patent hemostasis group, with 409 patients in each group. The recruitment number will be adjusted according to the trial progress and actual situation to ensure the statistical power of the study.

3.5.4 Compensation

Patients participating in this study will not receive any financial compensation for their participation.

4. Risk/Benefit Assessment

4.1 Benefits (Personal and Social Benefits)

4.1.1 Personal Benefits

Patients participating in the trial will have the opportunity to receive the latest medical technologies and treatment plans. For example, the balloon pressure monitoring method may improve hemostasis efficacy and reduce the risk of postoperative complications. Detailed examinations and monitoring during the trial will help detect potential health problems early and enhance the overall health management of patients.

4.1.2 Social Benefits

The trial results will provide important data for the optimization of treatment and hemostasis methods for patients undergoing coronary angiography in the Department of Cardiology, and may have a positive impact on clinical practice in the field of cardiology. By comparing the efficacy of the balloon pressure monitoring method with traditional hemostasis methods, the trial will help improve medical technologies, enhance patient treatment outcomes and quality of life. Ultimately, these research results will contribute to promoting the progress of cardiovascular disease treatment and reducing the related public health burden.

4.2 Risks

4.2.1 Potential Risks

(1) Operational risks: Complications that may be caused by the use of the balloon pressure monitoring method, including balloon pressure loss of control, vascular injury, etc.

(2) Postoperative complications: Patients may experience postoperative complications such as bleeding, hematoma, radial artery perforation, arteriovenous fistula, etc. Although the study aims to optimize hemostasis methods, these risks still require close monitoring and timely management.

(3) Individual differences: Different patients may have different tolerances to the balloon pressure monitoring method, especially elderly patients, pregnant women, and patients with severe underlying diseases, who may face a higher risk of complications.

(4) Data privacy risks: Patients' personal information and health data need to be strictly protected, and any data leakage may result in privacy violations.

(5) Ethical risks: It is necessary to ensure the adequacy of informed consent and patients' understanding of the study, especially in special populations. Any inadequate informed consent may lead to ethical issues.

(6) Trial risks: New unforeseen risks may be identified during the trial, requiring timely adjustment of the research protocol to ensure patient safety.

4.2.2 Risk Minimization Measures

(1) Strict medical operating procedures: Ensure that the use of the balloon pressure monitoring method complies with standardized operating procedures and is performed by specially trained medical personnel.

(2) Regular monitoring and assessment: Conduct regular health monitoring and assessment of patients during the trial to detect and address potential risks in a timely manner.

(3) Adequate informed consent: Before the start of the trial, fully explain all potential risks to patients and ensure they understand and agree to participate in the trial.

(4) Emergency response mechanism: Develop a detailed emergency response plan to ensure that prompt action can be taken in the event of adverse events to minimize the impact on patients.

4.3 Risks and Protection for Special Population Protection

4.3.1 Risks for Special Population Protection

(1) Elderly people: Elderly patients may have a higher risk of complications, such as cardiovascular diseases or multiple chronic diseases, and may have poor tolerance to new medical technologies.

(2) Pregnant and lactating women: This population may have special needs for medical measures in the trial and may experience adverse reactions to drugs or treatments.

(3) Patients with severe underlying diseases: These patients may have poor tolerance to trial interventions and a higher risk.

4.3.2 Protection Measures for Special Population Protection

(1) Strict screening and assessment: Conduct more rigorous screening and health assessment of special populations to ensure they meet the conditions for participating in the trial and can withstand the risks during the trial.

(2) Personalized risk management: Develop personalized risk management plans for special populations, including regular health checks and adjustment of treatment plans.

(3) Ethical review and consultation: Fully consider the needs of special populations in the trial design, obtain review and approval from the Ethics Committee, and provide appropriate consultation and support.

(4) Adequate communication and consent: Ensure that patients in special populations fully understand all relevant risks before participating in the trial, and provide detailed informed consent forms to ensure they participate voluntarily and can make informed decisions.

5. Ethical Issues in the Study

5.1 Informed Consent

Informed consent is a basic ethical principle in medical research. When recruiting participants, it is crucial to ensure they fully understand the purpose, process, potential risks, and benefits of the trial. The research team will provide detailed instructions, ensure patients have sufficient time for consultation, answer their questions, and voluntarily make a decision to participate. Special attention should be paid to the understanding ability and decision-making power of special populations (such as the elderly, pregnant women, and patients with severe underlying diseases).

5.2 Privacy Protection

During the trial, patients' personal information and health data must be strictly protected to comply with relevant laws and regulations on data privacy and protection. All participants' data should be anonymized, and sensitive information should not be accessible to unauthorized personnel. The research team will take necessary technical and management measures to ensure data security and confidentiality.

5.3 Balance of Risks and Benefits

During the study, it is necessary to ensure that the risks faced by participants are balanced with the potential benefits they may obtain. The study design should clearly assess the risk level and conduct strict monitoring. If adverse events or increased risks are observed during the trial, the research team must promptly adjust the research protocol to protect the safety of participants.

5.4 Fairness in Resource Allocation

During the recruitment process, all eligible patients should have a fair opportunity to participate. Discrimination in participation rights due to factors such as patients' socioeconomic status, ethnicity, or gender should be avoided. The research team will take proactive measures to ensure a transparent recruitment process and treat all potential participants fairly.

5.5 Handling of Adverse Events

When any trial-related adverse events occur, they must be recorded and reported immediately, and appropriate intervention measures should be taken. The research team should ensure that participants receive timely medical assistance and maintain continuous attention to their health status during the trial.

5.6 Ethical Review and Monitoring

Conduct reviews by the Institutional Ethics Committee (IEC) or Institutional Review Board (IRB), and perform regular monitoring throughout the study to ensure the research always complies with ethical standards and regulatory requirements.

6. Annual Plan

• September 2024 - May 2026: Complete the screening and enrollment of 818 patients. Hold a mid-year project team work report meeting to summarize problems encountered during the research, appropriately adjust the research content and progress, and further improve the research protocol.

• June 2026 - December 2026: Collate all data, conduct statistical analysis, and write papers and final reports.

Applicant's Commitment and Signature:

I guarantee the authenticity of the application content. I will fulfill the responsibilities of the project leader, strictly abide by the relevant national regulations on clinical research, effectively ensure the time for research work, conscientiously carry out the work, submit relevant materials on time, and consciously submit relevant reports during the research process in accordance with the requirements of the Ethics Committee's approval. If there is any misrepresentation or violation of regulations in the application, I will bear full responsibility.

Signature of the Principal Investigator:

Date:
