# Supplementary material for: Balloon pressure monitoring for radial artery hemostasis after transradial coronary procedures: protocol for a randomized controlled trial
Source: PLoS One. 2026 Jun 4;21(6):e0350563. doi: 10.1371/journal.pone.0350563 (PMC13235883; doi:10.1371/journal.pone.0350563)
Supplement: Attachment — Submitted filename: Response to Reviewers.docx [file pone.0350563.s003.docx]

Reviewer #1: Interesting paper. Some issues should be added

1) introduction should be shortened to no more than a page

2) is there a patent for this sytem?

3) open lable design is a limitation and should be added

4) kind of multiple imputation should be added (it should be overrealistic)

5) single center design limits generazibility

6) for sample size calculation data derive incidence of events from a study describing high volume center in optimal conditions. Please quote other papers

7) number of patients in each block shiuld be added

Response to Reviewer #1
We thank the reviewer for the insightful comments. We have addressed each point as follows and have revised the manuscript accordingly. All changes are highlighted in the revised manuscript using track changes.

Comment 1

introduction should be shortened to no more than a page

Response
We agree with the reviewer. The introduction has been substantially shortened to no more than one page. We have removed redundant content and streamlined the narrative while preserving all key background, evidence gaps, and study rationale.

Comment 2

is there a patent for this system?

Response
We appreciate your question regarding the patent status of the balloon pressure monitoring system. We have added a clear statement in the Competing interests section that the balloon pressure monitoring system described in this protocol is not protected by any patent. No patent is associated with this system.

Comment 3

open label design is a limitation and should be added

Response
We appreciate the reviewer’s careful comment. We would like to clarify that the open-label design has already been clearly recognized and fully discussed as an important limitation in the Study Limitations section of the manuscript. We have detailed the potential performance bias, Hawthorne effect, and related impacts introduced by the open-label design, together with the bias-mitigating strategies adopted. The relevant description has been maintained unchanged in the revised manuscript.

Comment 4

kind of multiple imputation should be added (it should be overrealistic)

Response

We thank the reviewer for the constructive suggestion. We have revised the handling of missing data to specify the type of multiple imputation (MICE with PMM) and added relevant details of the imputation strategy as recommended.

Comment 5

single center design limits generazibility

Response
We appreciate the reviewer’s comment. The limitation of the single-center design and its impact on generalizability has already been clearly stated and fully discussed in the Study Limitations section. The relevant content has been retained unchanged in the revised manuscript.

Comment 6

for sample size calculation data derive incidence of events from a study describing high volume center in optimal conditions. Please quote other papers

Response

We thank the reviewer for the valuable suggestion. We have supplemented the sample size justification with additional citations from a large meta‑analysis and an international consensus paper to support the control group event rate, while keeping all other parameters and calculations unchanged.

Comment 7

number of patients in each block should be added

Response
We apologize for the omission. We have now added the block size information in the randomization section. The sequence uses randomly varying block sizes of 4 and 6, which are concealed from investigators.

Reviewer #2: This manuscript presents a well-structured and methodologically sound trial protocol. The research question is relevant, and the study design is appropriate and clearly described. The level of detail provided ensures transparency and reproducibility.

Strengths:

The manuscript demonstrates several strengths, including a robust methodological framework, a clearly defined statistical analysis plan, and appropriate ethical oversight. The intervention and outcomes are well justified, and the protocol follows established reporting standards.

Comments:

I have no major concerns regarding the study design or reporting.

Response:

We greatly appreciate your positive feedback and constructive comments on our manuscript. We are pleased that you found the trial protocol well-structured, methodologically sound, transparent, and reproducible. All comments from the other reviewer have been carefully addressed and revised in the manuscript accordingly. Thank you again for your time and valuable input.
